# Optical Planar Waveguide Sensor with Integrated Digitally-Printed Light Coupling-in and Readout Elements

**DOI:** 10.3390/s19132856

**Published:** 2019-06-27

**Authors:** Jorge Alamán, María López-Valdeolivas, Raquel Alicante, Carlos Sánchez-Somolinos

**Affiliations:** 1 Instituto de Ciencia de Materiales de Aragón (ICMA), CSIC-Universidad de Zaragoza, Departamento de Física de la Materia Condensada, 50009 Zaragoza, Spain; 2 BSH Electrodomésticos España, S.A., Polígono Industrial de PLA-ZA, Ronda del Canal Imperial de Aragón, 50197 Zaragoza, Spain; 3 Centro de Investigación Biomédica en Red de Bioingeniería, Biomateriales y Nanomedicina (CIBER-BBN), C Mariano Esquillor s.n., 50018 Zaragoza, Spain

**Keywords:** integrated optical planar waveguide sensors, flexible sensors, organic-inorganic hybrid materials, liquid crystalline thermoresponsive materials, inkjet printing, luminescent materials

## Abstract

Optical planar waveguide sensors, able to detect and process information from the environment in a fast, cost-effective, and remote fashion, are of great interest currently in different application areas including security, metrology, automotive, aerospace, consumer electronics, energy, environment, or health. Integration of networks of these systems together with other optical elements, such as light sources, readout, or detection systems, in a planar waveguide geometry is greatly demanded towards more compact, portable, and versatile sensing platforms. Herein, we report an optical temperature sensor with a planar waveguide architecture integrating inkjet-printed luminescent light coupling-in and readout elements with matched emission and excitation. The first luminescent element, when illuminated with light in its absorption band, emits light that is partially coupled into the propagation modes of the planar waveguide. Remote excitation of this element can be performed without the need for special alignment of the light source. A thermoresponsive liquid crystal-based film regulates the amount of light coupled out from the planar waveguide at the sensing location. The second luminescent element partly absorbs the waveguided light that reaches its location and emits at longer wavelengths, serving as a temperature readout element through luminescence intensity measurements. Overall, the ability of inkjet technology to digitally print luminescent elements demonstrates great potential for the integration and miniaturization of light coupling-in and readout elements in optical planar waveguide sensing platforms.

## 1. Introduction

Sensors are often described as platforms able to detect information, for example temperature, pressure, humidity, or the presence of chemical compounds, from the environment and transform this information into different signals. Most common sensors usually respond to an external stimulus with an electrical signal that is later transmitted and processed by other electronic systems to which they are interfaced. The language spoken between sensors and devices in these cases is therefore based on the flux of electrons; however, the flux of photons can similarly be used to transfer information in sensing platforms. Optical sensors present attractive features when compared with electrical ones, since, for example, they are not subjected to electromagnetic interference, so they can be used in a harsh environment with high voltages or intense magnetic fields. Besides, the possibility to transfer information through optical waveguides enables the generation of sensor networks that can be connected and monitored remotely without the need to transform the detected information into an electrical signal at each sensing site. The use of light with different properties (e.g., wavelength) also provides the possibility to multiplex several signals in a single waveguide easily. Even more, the employed waveguides can be lightweight, flexible, and inexpensive. All these characteristics make optical sensing platforms of great interest in different application areas including metrology, quality and process control, security, or (bio)medicine [1,2,3,4,5].

An optical sensor typically entails different components such as photonic sources to generate the probing light, optical elements to condition and direct this light towards the sensing region, as well as readout devices to obtain the analysis results. In this field, optical fiber sensors have reached a high degree of development. Coupling of light from lasers or light-emitting diodes (LEDs) is routinely performed currently by means of optical fiber couplers, and progress in sensing materials has enabled the implementation of sensors with improved sensitivity, portability, and reduced size [6,7]. 

Besides sensors relying on optical fibers, optical sensing systems integrated in planar waveguide geometries have also been extensively explored as they can integrate networks of sensing elements over large areas [8,9,10]. In these planar systems, it is highly desirable to miniaturize and integrate optical elements of the sensing platform such as detection and readout systems or light sources in the planar waveguide [11,12,13,14]. Moreover, for application areas such as biomedicine, microfluidics, or electronic skin, other specific requirements such as the compatibility with materials of different natures (e.g., glass, polymer, or metal), conformability, bendability, or stretchability need to be properly addressed to contribute to the overall sensing device performance [15,16,17,18]. Besides, the use of easily scalable, flexible, and reliable high-throughput fabrication techniques to synergistically combine photonic and sensing elements in thin films is required for the further development of these technologies. In this direction, a large effort is currently being made towards the development of materials and compatible manufacturing technologies enabling the large-scale, high-speed, and low-cost production and preparation of microoptical elements, as well as planar photonic systems on polymer foils. Waveguides have been generated in flexible substrates through, for example, lamination or inkjet-printing methods [19,20,21,22,23,24,25]. Interestingly, luminescent acrylate formulations have been applied through inkjet printing on polymeric waveguides generated on poly(methyl methacrylate) (PMMA) substrates by Bollgruen and coworkers to create remotely-addressable light sources that can couple light into flexible waveguides [26]. Moreover, regarding the sensing method, it is well known that the use of luminescence intensity measurements to perform magnitude readout advantageously leads to sensitivity improvement when there is no overlap between the exciting and emitted light spectra [27]. Even more, in a waveguide geometry, the light not absorbed in the readout element can be guided away from the detection region, further facilitating discrimination of the luminescence from the excitation light, leading to improved quality of the detectable signal.

**Figure 1:**
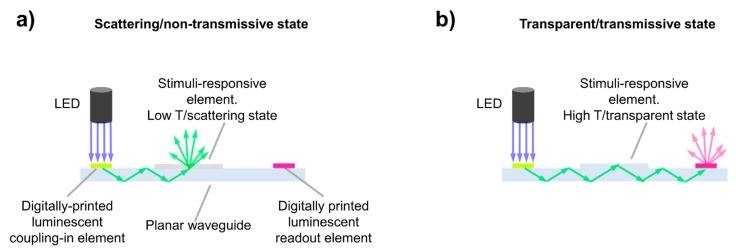
Optical planar waveguide sensor with integrated digitally-printed luminescent light coupling-in and readout elements. Remote excitation of the first luminescent element, by using an LED for example, enables coupling of light into the waveguide. Light is guided towards the thermoresponsive LCP element that regulates the light intensity transmitted towards the end of the waveguide where the second luminescent element is placed. (**a**) Scattering/non-transmissive state, at low temperature: light is scattered out of the waveguide at the stimuli-responsive element. (**b**) Transparent/transmissive state, at high temperature: light is not scattered at the stimuli-responsive element, and therefore, light further propagates in the waveguide and reaches the second luminescent layer. Remote luminescence intensity measurements allow quantifying the temperature in the sensor range.

In this paper, we present an optical temperature sensor having a planar waveguide architecture and including two luminescent layers with matched emission properties. These two emissive elements allow integrating light coupling-in and readout functions in the planar waveguide structure (Figure 1). Illumination of the first luminescent element with light in its absorption band induces emission of light that is partially coupled into the guided modes of the planar waveguide. Excitation of this element can be remotely performed without the need for special alignment of the light source. As temperature sensing materials, we include liquid crystalline polymers (LCP). The changes of molecular order in these systems, in response to temperature variations, make them suitable materials for this purpose [28,29,30,31]. Changes in light scattering of the liquid crystalline (LC) polymeric film with temperature enable the regulation of the light coupled out from the waveguide at this location. Besides temperature sensing, used in this paper as a simple example, LC materials could be tailored to be responsive to other stimuli such as moisture, pH, or chemical compounds as well [32,33,34,35,36,37]. After passing the region of the sensing element, light remaining in the waveguide reaches the second luminescent element. The emission wavelength range of the first element is chosen to overlap with the excitation band of the second one, so the light generated in the first element, which is partially coupled into the waveguide, serves as excitation for the second element that emits light at longer wavelengths. Temperature readout can be carried out by measuring the luminescence intensity of this element. For the implementation of the two luminescent functional layers, we make use of our recently-developed inkjet printing platform based on photoacid-catalyzed organic-inorganic hybrid formulations including light-emissive molecules [24,38]. Inkjet printing, enabling the digital deposition of materials, has been demonstrated to be a powerful tool for the preparation and integration of functional elements and devices in a flat geometry [25,39,40,41,42,43,44,45,46]. In our case, inkjet printing technology allows the integration in planar waveguide structures of light coupling-in and readout elements. With the presented architecture, photonic elements such as light sources or detectors can be physically separated from the sensing device, still allowing remote and easy light coupling-in and readout; and therefore, the use of the sensor in adverse environments with, for example, extreme humidity or high electrical or magnetic fields. The implementation of the sensors in rigid and flexible waveguides is also demonstrated.

## 2. Materials and Methods

### 2.1. Planar Waveguides

Cleaning procedure: Microscope glass slides (Thermo ScientificTM SuperFrost©, extra-white soda-lime glass, dimensions: 76 mm × 26 mm, 1 mm thick, n = 1.517 at 546 nm) were employed as planar waveguides in some of the experiments. Pre-cleaning of these glass slides using soapy water was carried out by gently rubbing the glass surface, using nitrile gloves. The slides were rinsed with water and introduced in an ultrasonic bath with soapy water for 10 min. After this, the glass slides were refluxed with Milli-Q water and ultrasonicated again in Milli-Q water for 10 min. The slides were then flushed with isopropyl alcohol. A third ultrasonic bath was carried out, in this case with isopropyl alcohol for 10 min. Finally, the substrates were dried with compressed air.

Cyclic olefin polymer (COP) foil from Zeonor (188 μm-thick polymer foil, n = 1.53 at 589 nm, Dusseldorf, Germany) and microscope COP slides, supplied from BeonCHIP (Zaragoza, Spain), were used as polymeric waveguides. COP rigid slides were pre-cleaned and were used as received. The flexible foil substrate was provided with a protective foil that was removed just before processing.

### 2.2. Substrate Treatments

UV ozone treatment was performed on a UV ozone reactor UVO 342 (Jelight company Inc., Irvine, CA, USA) to remove any contamination in the glass substrates, leaving silanol groups exposed on the surface [38]. As a result, important changes in the wettability and in the adhesion were obtained [24].

### 2.3. Surface Functionalization with Fluorosilane

The glass substrates, cleaned as described above, and UV ozone treated for 1 hour, were placed in a desiccator containing a glass slide with 50 μL of 1H,1H,2H,2H-perfluorooctyltrichlorosilane (PFOTClS) from Aldrich. A vacuum was applied in the desiccator until a pressure of 100 mbar was achieved. After 30 min, the substrates were taken out, thoroughly rinsed with isopropyl alcohol, dried with compressed air, and heated for 10 min at 110 °C in air [47].

### 2.4. Temperature-Responsive Liquid Crystalline Material

6-(4-Cyano-biphenyl-4′-yloxy)hexyl acrylate (A6OCB, also known as RM 105), a pro-mesogenic molecule functionalized with one reactive acrylate group, was acquired from Synthon GmbH under the reference STO3474. A6OCB presents a crystalline phase at room temperature (RT) and melts at 70–71 °C. 1,4-Bis-[4-(6-acryloyloxyhex-yloxy)benzoyloxy]-2-methylbenzene (RM82) from Merck is a mesogen functionalized with two acrylate end groups. 1-Hydroxycyclohexyl phenyl ketone, also known as Irgacure 184 (IRG184), was used as a UV photoinitiator. A mixture comprising 99 wt% of A6OCB and 1 wt% of RM82 was prepared as a precursor of the thermoresponsive LCP. Besides, 3 wt% of IRG184 photoinitiator was added. To help mixture homogenization, dichloromethane (CH_2_Cl_2_) in an equivalent wt% to the weight of the solid material was added. The solids immediately dissolved just after a mild shaking of the containing flask. Afterwards, the flask remained open overnight in a fume hood, and the weight was controlled the day after, confirming that all the dichloromethane had been eliminated from the mixture.

### 2.5. Ink Materials

The following materials were used for the formulation of the inks: 3-glycidoxypropyltrimethoxysilane (GPTMS), a hybrid organic-inorganic monomer bearing an epoxy and a trialkoxysilane group purchased from Alfa Aesar; the epoxy resin Epikote 157, with an average of eight aromatic benzene rings and eight epoxide reactive groups, acquired from Momentive; dimethoxydiphenylsilane (dPDMS), a disilane monomer with two aromatic rings, supplied by Aldrich; a photoacid generator (PAG), triarylsulfonium hexafluorophosphate salt (50% in propylene carbonate), from Aldrich that, upon excitation with UV actinic light, triggers the polymerization reaction of the organic epoxides and, concurrently, catalyzes the hydrolysis and condensation of the alkoxide groups; besides, BYK-333, a polyether-modified polydimethylsiloxane from BYK Chemie, was used to regulate the surface tension of the inks and to promote surface wetting. 

To provide the inks with luminescent properties, two different dyes were employed: (i) fluorescein 27 (F27), a luminescent dye having an emission centered at 520 nm (green light) [48,49]; and (ii) Rhodamine B, which strongly absorbs in the green and emits light in the orange-red region of the spectrum, centered at 585 nm [50]. F27 and Rhodamine B were purchased from Lambda Physic under the references Lambdachrome LC 5530 and LC6100, respectively. All the materials were used as received.

### 2.6. Ink Preparation

The two photopolymerizable luminescent formulations employed in this work were based on the addition of a small amount of emissive dye to a jettable ink, previously developed in our laboratory, incorporating different monomers. Namely, 50 wt% of GPTMS, 25 wt% of Epikote 157, and 25 wt% of dPDMS were the components of the basic formulation. To the weight of these reactive monomers, a percentage of 2 wt% of triarylsulfonium hexafluorophosphate salts was added to the mixture. Additionally, 0.05 wt% of BYK-333 was added to improve the wetting of the inks in the substrate [51] and to introduce viscoelasticity in the inks [24]. The first luminescent ink, named HRI-F27-02, contained fluorescein 27 (F27), and it was based on the abovementioned basic formulation [24]. Then, 0.2 wt% of F27 was added to the total weight to provide the printed layers with luminescence in the region of 520 nm upon excitation with light in the UV or blue regions. The second luminescent formulation, containing Rhodamine B and named HRI-RhodB-02, had the same composition except for the luminescent dye. To provide HRI-RhodB-02 with orange-red luminescence when excited in the green, 0.2 wt% of Rhodamine B was added to the basic formulation. 

### 2.7. Inkjet Printing

Inkjet printing was carried out using a custom-made inkjet printer system (In-2 Printing Solutions, Navarra, Spain) with Xaar-126/80 piezoelectric printheads (Xaar, Cambridge, U.K.). The printhead had 126 nozzles (50 μm in diameter) arranged in a line with a distance of 137 μm between them. The line of nozzles was perpendicular to the direction of the substrate motion, which moved along a line under the fixed printhead. As a result, the vertical resolution (in the direction of the line of nozzles, perpendicular to the substrate motion) was 185 dots per inch (dpi). The horizontal resolution (in the direction of the substrate movement) depended on the firing frequency and the relative speed of the substrate with respect to the printhead. The printhead was commanded by a Xaar XUSB drive electronics, controlled with a PC. The software (from Xaar) enables the control of the parameters of the printhead, synchronization of the different elements, and transfer of patterns to be printed (bitmap file) to the printhead. The movement of the substrate while printing was at a constant speed of 20 mm/s by using an eTrack linear stage from Newmark systems Inc. (Mission Viejo, CA, USA) commanded by IMS-Terminal software (Marlborough, MO, USA). The printhead was mounted in a metallic block, provided with a heater and thermocouple connected to a temperature control unit that fixed the printhead temperature at the set point (32 °C) [24,38].

### 2.8. Thermoresponsive LCP Film Preparation

The photopolymerizable mixture together with spherical silica spacers (50 µm) was applied on top of the waveguide plate. The waveguide was heated at 75 °C, and the photopolymerizable mixture was molten at this temperature. A fluorinated glass plate (previously heated to 75 °C) was applied and pressed on top of the photopolymerizable mixture provided with spacers that defined the thickness of the final film. Careful application of this glass cover resulted in a liquid layer free of bubbles ready for photopolymerization. A UV lamp Exfo OmniCure S2000 UV (Gentec, Nivelles, Belgium) was used with a UV bandpass filter (wavelength range of 320–390 nm) for this purpose. Photocuring of the photopolymerizable mixture was carried out by heating the sample at 75 °C and then exposing to UV light with a power of 140 mW/cm^2^ for 30 s. A subsequent post-curing step was carried out by exposing the film with a power of 10 mW/cm^2^ for 5 min. The fluorinated glass was carefully removed, leaving the photopolymerized film in contact with the air. 

### 2.9. Ink Photocuring

For the UV photopolymerizable inks, after inkjet deposition, the samples were exposed to UV light, using the same light source as in the thermoresponsive curing, with a power of 10 mW/cm^2^ for 5 min. The curing process was carried out under mild vacuum conditions placing the printed samples inside a chamber with an optical access. A vacuum of 100 mBar was attained inside the chamber by using a vacuum pump [38]. To reduce the evaporation of the components of the ink while the vacuum reach the desired pressure, UV exposure was immediately activated once this pressure level was reached. 

### 2.10. Characterization

Luminescence of the deposited films was characterized using a Perkin Elmer LS 50B spectrometer. Polarization optical microscope (POM) images of the liquid crystalline textures were taken using an optical microscope OLYMPUS Eclipse i80 provided with crossed polarizers. For temperature-dependent observations, the microscope was equipped with a Linkam LTSE420 heating stage. 

Thermogravimetric analysis (TGA) was performed using a Netzsch TG 209 Libra F1 Instrument to obtain the temperature of the onset of the decomposition weight loss curve and the maximum of the derivative of the TGA curve. 

## 3. Results

**Figure 2:**
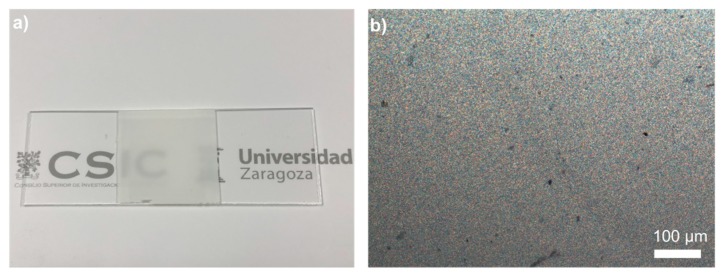
(**a**) Photograph of a 50 µm-thick sample of LCP (26 mm × 26 mm) applied in the central part of a glass microscope slide (76 mm × 26 mm). The LCP film shows high turbidity at RT when compared to the bare glass (at the two sides of the LCP). (**b**) POM image of an LCP thin film sample at RT.

Prior to integrating, through digital inkjet printing, the light coupling-in and readout luminescent elements depicted in Figure 1, we integrated, as a first step, the temperature sensing element in a planar waveguide geometry. As mentioned in the Introduction, a stimuli-responsive LCP element that changes its scattering properties in response to temperature was incorporated into the central part of the planar waveguide structure. A microscope glass slide was initially used as the planar waveguide structure in this work. This type of simple thick planar waveguide has been used for example in the implementation of different biosensing devices [9]; however, the different described elements, thermoresponsive and luminescent films, could be easily integrated in more sophisticated planar waveguiding structures. The thin layer of LCP presented a highly scattering morphology at low temperature and a transition to an isotropic state at higher temperature, at the clearing point (Tc). To apply this layer on top of the waveguide, first, a photopolymerizable mixture comprising 99 wt% of A6OCB monoacrylate and 1 wt% of RM82 diacrylate was prepared. The monofunctional promesogenic monomer A6OCB was chosen as the main component of the mixture since the resultant side chain liquid crystal polymer (SCLCP), obtained after radical polymerization, presented liquid crystalline behavior [52]. The small amount of mesogenic diacrylate added to the mixture was incorporated to stabilize the resultant film and to hinder dewetting upon heating at high temperature. To enable photopolymerization, a small amount (3 wt%) of UV photoinitiator was added. The prepolymer mixture presented a transition from the crystalline solid to an isotropic liquid at 70 °C, as determined by POM, essentially at the same temperature as the majority monomer component A6OCB. A thin film with a fixed square-shape geometry of the photopolymerizable mixture was applied on top of the waveguide, in its central part, as schematically shown in Figure 1. Film thickness was fixed by using 50-μm spacers dispersed in the mixture and a second fluorinated glass plate, as described in the Section 2. Exposure at 75 °C to UV light led to a noticeable change in the transmission properties of the material. The sample, which was transparent at 75 °C before UV exposure, became turbid in seconds just after actinic light was switched on, keeping the sample at a constant temperature of 75 °C. Excitation of the photoinitiator with UV light produced radicals that initiated the chain growth polymerization of the acrylate monomers. Once the photocuring process (see the Section 2) was completed, the sample was cooled down to RT keeping the same turbid appearance (see Figure 2a), and the fluorinated glass was carefully removed, leaving a highly scattering solid polymeric film attached to the waveguide.

As mentioned before, cyanobiphenyl acrylate monomers tend to lead to polymers showing liquid crystalline behavior [52]. POM observation of a thin LCP film as a function of temperature revealed a birefringent grainy texture in the cured samples at the curing temperature (75 °C), as shown in Figure 2b. The birefringent texture, observed at the POM, totally disappeared when heating at 134 °C, at which isotropization takes place. Cooling down below the clearing point recovered essentially the same birefringent fine grainy texture as the as-polymerized film. This recovery was ascribed to the slightly cross-linked nature of the system. The presence of crosslinking sites provided memory to the system, typical of LC elastomers, when it was heated above its isotropization temperature and then cooled back to the mesophase range. The heating and cooling process was repeated several times (>20 times), showing the same optical qualitative behavior with no performance degradation. Indeed, the material showed good thermal stability at temperatures well above this transition temperature to the isotropic state, as assessed by TGA, giving an onset of the decomposition weight loss curve at temperatures above 300 °C (see Figure S1 in the Supplementary Information).

**Figure 3:**
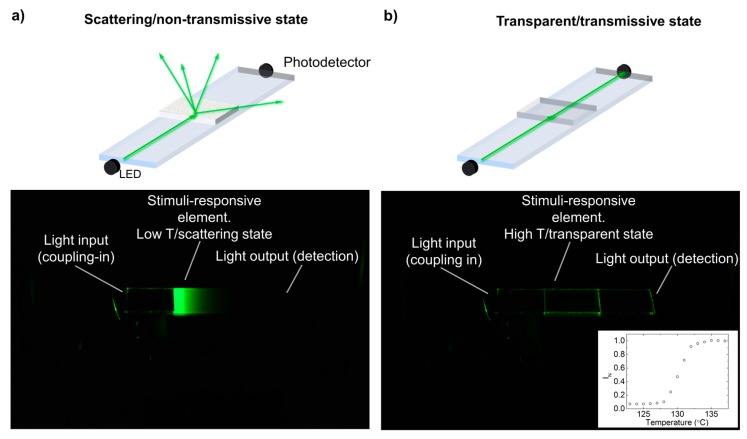
(**a**) and (**b**) Schematic representation (top) and photograph (bottom) of the optical planar waveguide sensor using a green LED (peak wavelength of 520 nm) to couple light into the waveguide at one of the waveguide extremes and a photodetector to measure transmitted light at the other extreme (**a**) in the scattering/non-transmissive state, at low temperature, and (**b**) in the transparent/transmissive state, at high temperature. The inset in (**b**) shows a plot of the green light intensity measured at the detection edge of the waveguide as a function of the temperature of the sensing area.

In order to characterize the temperature dependence of the light transmission of the constructed thermosensitive waveguide, the sensing area, provided with the LCP layer, was placed on top of a temperature-controlled metallic block. In this simplified configuration (Figure 3a,b), light was coupled into the waveguide by placing an LED (520-nm peak wavelength) at the edge of the waveguide. This is an efficient way to couple energy into waveguides that can be even further optimized by the use of lenses or reflectors at the LED-waveguide edge interface [9]. A Si photodetector was placed at the other extreme of the planar waveguide, enabling quantification of the light reaching this side. The inset of Figure 3b shows a plot of the light intensity measured at the edge of the waveguide as a function of the temperature of the sensing region. It can be seen that the level of light reaching the detector remained low at temperatures below 126 °C and gradually increased as temperatures went above this temperature. At temperatures below 126 °C, within the liquid crystal mesophase, the light travelling from the LED through the waveguide reached the highly scattering LCP film, coupling out light, as can be identified by its green glowing appearance (see Figure 3a). Glow strongly attenuates from left to right in the image, showing the efficiency of the LCP extracting light from the waveguide. As temperature increased from 126–134 °C, the intensity of transmitted light gradually increased as the scattering of the LCP layer progressively decreased. At temperatures above 134 °C, the LCP layer became transparent (see Figure 3b) and light remained waveguided, reaching the waveguide extreme. The large ratio between the light intensity measured at the high and the low temperature states demonstrated that only a small portion of the light coupled into the waveguide modes reached the extreme of the planar waveguide in the low-temperature, high-scattering state of the LCP layer, demonstrating its good performance as a thermo-optical valve.

Trying to simplify the injection of light into the waveguided modes, the incorporation of an alternative light coupling-in system was attempted in the waveguide sensor structure. Traditional methods to couple light into a planar waveguide include the incorporation of diffractive gratings or the use of coupling prisms in combination with lasers [53]. Light can also be coupled by the incorporation of luminescent entities (organic dyes, phosphors, quantum dots, etc.) in the waveguide structure [54,55]. Light with a wavelength matching the absorption band of the luminescent layer can be absorbed when passing through it. The absorbed light can be re-emitted at longer wavelengths in all directions. Part of this emitted light is trapped in the waveguided structure by total internal reflection (TIR), at the waveguide-air interface. Emission light rays travelling through the waveguide and reaching this interface at an angle above the critical angle θ_c_ for TIR (θ_c_ = n_s_/n_air_; being n_s_ and n_air_ the refractive indexes of substrate and air, respectively) cannot propagate in the air. As a result, energy remained confined in the waveguide, that is, this emitted light was coupled into its propagation modes. This principle has also been recently used by Bollgruen et al. to couple light in channel waveguides in plastic foils [26]. Despite being less efficient, in terms of energy coupled, in comparison to grating and prism coupling systems, luminescent couplers constitute a simple way to implement and a simple-to-use principle without the need for complex optical alignment systems or specific light sources (e.g., lasers). The use of sunlight as the excitation light source is even possible. 

**Figure 4:**
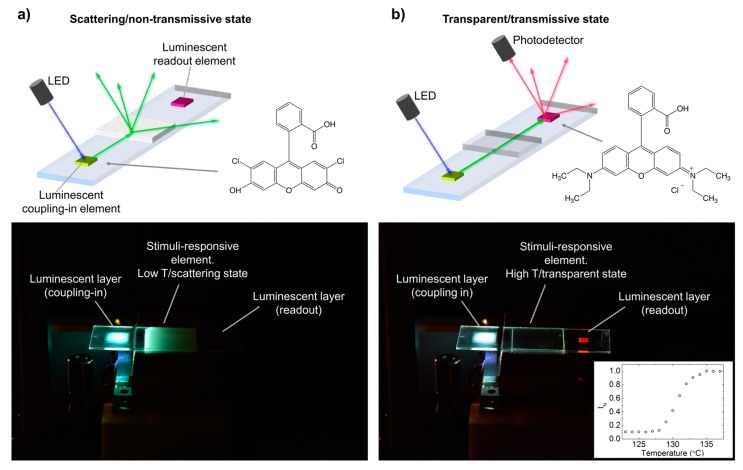
(**a**,**b**) Schematic representation (top) and photograph (bottom) of the optical planar waveguide sensor including a luminescent coupling element and a luminescent temperature readout element (**a**) in the scattering/non-transmissive state, at low temperature, and (**b**) in the transmissive state, at high temperature. Light from a UV LED (peak wavelength at 365 nm) excites from the top the photocured HRI-F27-02 layer that emits light, partly coupled in the planar waveguide and travelling towards the thermoresponsive liquid crystal polymer sensor material. Waveguided light reaching the HRI-RhodB-02 layer is partly absorbed and re-emitted as orange-red light. A photodetector measures the red light (633 nm) intensity emitted by this luminescent layer as a function of the LCP layer temperature. The molecular structures of F27 and RhodB are shown in (**a**) and (**b**), respectively. The inset in (**b**) shows a plot of the emitted red light (633 nm) measured as a function of temperature at the sensing region.

We made use of this principle by incorporating, through digital inkjet printing, a luminescent layer that acted as a light coupling-in element for the waveguide when remotely excited with light of the appropriate wavelength (see Figure 4). Besides, the digital character of inkjet deposition enabled precise placement of the light coupling-in element. To implement this, a luminescent layer of a hybrid organic-inorganic material containing the F27 chromophore (HRI-F27-02) was applied in one side of the planar waveguide sensor structure. This dye (molecular structure shown in Figure 4a) emitted green light when excited at shorter wavelengths as shown in the emission spectrum of Figure S2 in the Supplementary Information. The emitted light was partly coupled into the waveguide structure propagating towards the thermo-optical sensor. Printing of these continuous areas was achieved with our printer by using 2880 dpi in the substrate moving direction, while keeping 185 dpi in the perpendicular direction. These printing conditions resulted in drop coalescence leading to continuous features in the substrate, as previously described elsewhere [38]. Besides the luminescent light coupling-in element, we incorporated a second inkjet-printed luminescent layer (HRI-RhodB-02) on the other side of the planar waveguide sensor structure. This second element contained Rhodamine B (molecular structure shown in Figure 4b), a dye that absorbs in the green region of the spectra and emits orange-red light, as shown in the emission spectrum of Figure S3 in the Supplementary Information. The light emitted by this element was partly coupled out into the air, becoming visible and therefore acting as a luminescent temperature readout enabling remote sensing. As in the case of the light coupling-in element, this second mark can also be digitally structured, as we have previously demonstrated [38]. 

In this case, light was coupled into the planar waveguide structure by exciting, with UV light coming from an LED (Thorlabs M362L2-C1, peak wavelength at 365 nm, spectrum of reference [56] placed on top), the HRI-F27-02 layer previously deposited on one side of the waveguide. Part of the coupled light propagated and reached the thermo-optical element that regulated, as a function of temperature, the light passing to the other extreme of the planar waveguide structure where the mark made with HRI-RhodB-02 was placed. The intensity of the luminescence of this HRI-RhodB-02 mark (measured at 633 nm by using a Si photodetector provided with an interferential filter with 10-nm FWMH) as a function of temperature of the thermo-optical layer is presented in the inset of Figure 4b. A low level of red emitted light was measured at temperatures below 126 °C, temperatures at which the LCP was in the high scattering state. The light intensity increased as temperature went above 126 °C and reached a plateau level at temperatures higher than 134 °C. As in the previously-described configuration, a sufficient ratio between the light intensity at the high and the low temperature of around 10 was measured, demonstrating the suitability of the coupling and the readout abilities using the luminescence to implement the temperature optical planar sensor. A qualitatively similar behavior was observed when excitation of the HRI-F27-02 layer was done with blue light of 455 nm, instead of using UV light (see Figure S4 in the Supplementary Information). 

**Figure 5:**
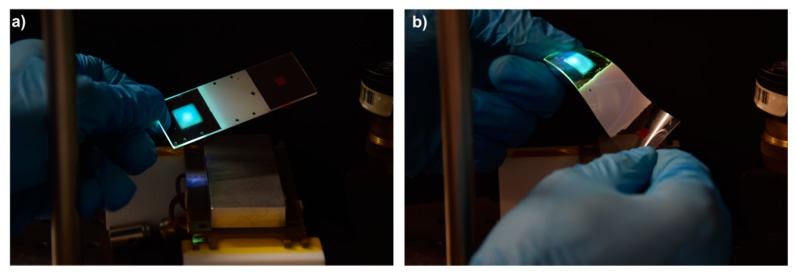
(**a**) Optical planar waveguide sensor including a luminescent coupling element and a luminescent temperature readout element fabricated on (**a**) a rigid cyclic olefin polymer (COP) substrate and (**b**) a flexible COP thin sheet. The sensor, at RT, is in the low T/high scattering state, and the luminescent coupling element is excited with a UV LED placed on top.

The employed photoacid-catalyzed luminescent inks were demonstrated to be very versatile in terms of their compatibility with different types of substrates [38]. As mentioned, printing on polymeric substrates such as poly(ethylene terephthalate) (PET), acrylonitrile butadiene styrene (ABS), or polycarbonate (PC) has been demonstrated to lead to thin layers of hybrid material showing good adhesion, as well as mechanically flexible. Trying to expand the scope of the present study and show the possibilities of the disclosed sensor, we have explored its implementation in substrates different from glass. As a proof of principle, the sensor was built on a cyclic olefin copolymer (COP) substrate that acts as a planar waveguide. Inkjet printing of the luminescent materials was directly carried out in the as-cleaned substrates without further treatment. Similarly, as done with the glass substrates, a continuous area of luminescent ink containing F27 dye was applied and cured. Excitation of this layer with UV light coupled green light into the COP waveguide. The luminescent indicator printed using the Rhodamine B-containing ink was digitally applied and cured. The thermoresponsive liquid crystal mixture was applied and cured using a similar protocol to that used in the glass-based sensors. A qualitatively similar response was obtained in this prototype compared to that observed in glass. Both a rigid COP waveguide (1.2 mm thick) and a flexible version of the sensor, based on a thin film of COP that can be easily conformed to sense curved surfaces, were generated, as shown in Figure 5.

## 4. Conclusions

An optical planar waveguide temperature sensor integrating digitally-printed luminescent light coupling-in and readout elements in the waveguide was presented. The remote excitation of the first luminescent element enabled the coupling of light into the planar waveguide, without the need for special alignment of the light source. When waveguided light propagated through the sensing region, a thermoresponsive LCP regulated the amount of light reaching the readout region. By matching the emission and excitation properties of the luminescent light coupling-in and readout elements, respectively, quantification of the temperature of the sensing region can be carried out through simple remote luminescence intensity measurements of the readout element. The digital character of inkjet technology, allowing the selective deposition of luminescent elements at precise locations and with well-defined geometries, offers great potential for the integration of light coupling-in and miniaturized readout luminescent elements in optical planar waveguide sensors. The hybrid organic-inorganic nature of the photoacid catalyzed formulations employed in the luminescent inks has led to deposits with excellent adhesion to substrates of different natures such as rigid glass sheets or flexible COP thin films. Despite the range of temperature of the presented sensor, around 130 °C, being narrow, by using other thermoresponsive LC materials, sensors with tailored properties and other ranges of sensing temperature could be generated using the same architecture. As a result, the fabrication platform, which included materials and processing techniques, can lead to flexible, compact, and robust digital integration of different elements to generate fully-reversible, cost-effective, portable, and versatile sensing devices. The prepared sensor, based on luminescence, can be remotely addressed and read using external photonic elements without complex optical alignment, therefore having great potential in diverse application areas including electronics, automotive, aerospace, environment, or health.

## Supplementary Materials

Supplementary materials can be found at https://www.mdpi.com/1424-8220/19/13/2856/s1. Figure S1: TGA analysis and derivative of the TGA curve for the photocured LCP, Figure S2: Photoluminescence emission spectrum of a deposited film of HRI-F27-02 cured under mild vacuum conditions. Photoluminescence emission spectrum is taken with excitation at 390 nm, Figure S3: Photoluminescence emission spectrum of a deposited film of HRI-RhodB-02 cured under mild vacuum conditions. Photoluminescence emission spectrum is taken with excitation at 530 nm, Figure S4: Optical planar waveguide sensor including a luminescent coupling element and a luminescent temperature readout element (a) in the non-transmissive state, at low temperature, and (b) in the transmissive state, at high temperature. Light from a blue LED (peak wavelength at 455 nm), excites the photocured HRI-F27-02 layer that emits light, partly coupled in the planar waveguide and travelling towards the thermoresponsive liquid crystal polymer sensor material. Waveguided light reaching the HRI-RhodB-02 layer is partly absorbed and re-emited as orange-red light. A photodetector measures the red light (632 nm) intensity emitted by this emissive layer as a function of the LCP layer temperature.
