# Supplementary material for: Optical Planar Waveguide Sensor with Integrated Digitally-Printed Light Coupling-in and Readout Elements
Source: Sensors (Basel). 2019 Jun 27;19(13):2856. doi: 10.3390/s19132856 (PMC6651219; doi:10.3390/s19132856)
Supplement: Supplementary file 1 [file sensors-19-02856-s001.pdf]

sensors

# Optical Planar Waveguide Sensor with Integrated Digitally-Printed Light Coupling-in and Readout Elements

**Jorge Alamán [1,2], María López-Valdeolivas [1], Raquel Alicante [1] and Carlos Sánchez-Somolinos [1,3,*]**

[1] Instituto de Ciencia de Materiales de Aragón (ICMA), CSIC-Universidad de Zaragoza, Departamento de Física de la Materia Condensada, Zaragoza 50009, Spain; m_lopez@unizar.es (M.L.-V.); raquela@unizar.es (R.A.)

[2] BSH Electrodomésticos España, S.A., Polígono Industrial de PLA-ZA, Ronda del Canal Imperial de Aragón, 50197 Zaragoza, Spain; jorge.alaman@bshg.com

[3] Centro de Investigación Biomédica en Red de Bioingeniería, Biomateriales y Nanomedicina (CIBER-BBN), C Mariano Esquillor s.n., Zaragoza 50018, Spain

* Correspondence author: carlos.s@csic.es; Tel.: +34-8765-53770

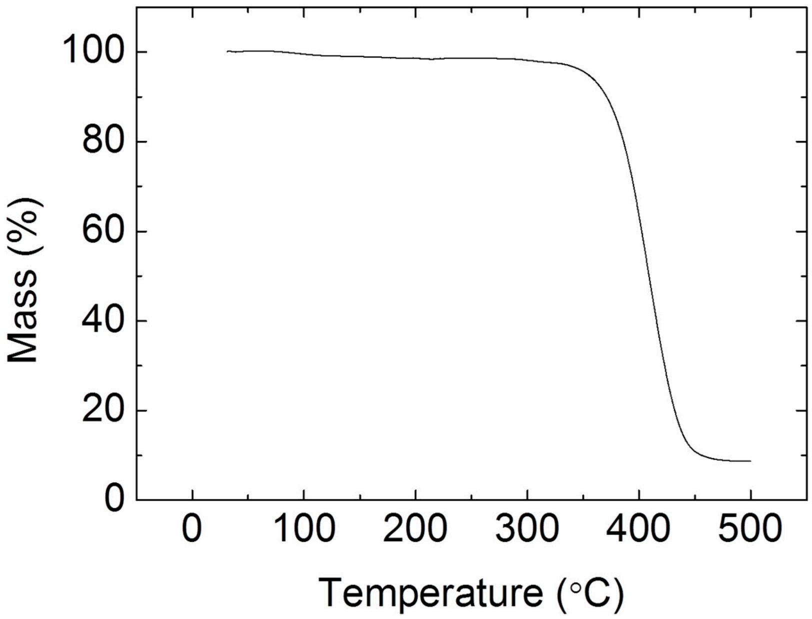


**Figure S1.** TGA analysis and derivative of the TGA curve for the photocured LCP.

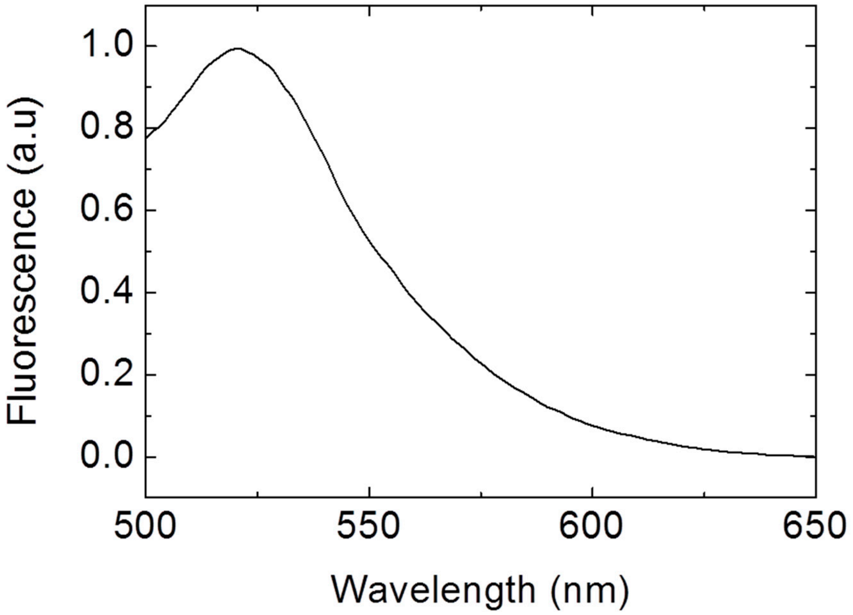


**Figure S2.** Photoluminescence emission spectrum of a deposited film of HRI-F27-02 cured under mild vacuum conditions. Photoluminescence emission spectrum is taken with excitation at 390 nm.

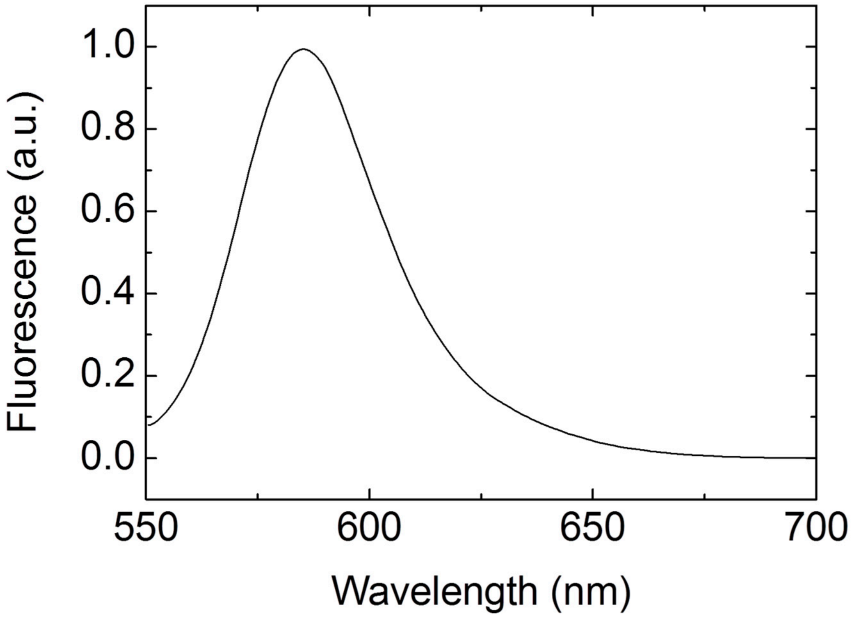


**Figure S3.** Photoluminescence emission spectrum of a deposited film of HRI-RhodB-02 cured under mild vacuum conditions. Photoluminescence emission spectrum is taken with excitation at 530 nm.

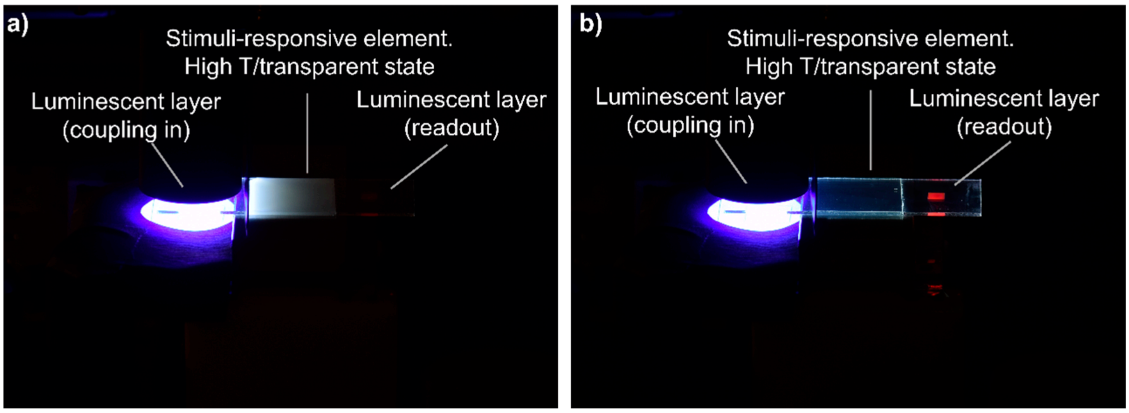


**Figure S4.** Optical planar waveguide sensor including a luminescent coupling element and a luminescent temperature readout element a) in the non-transmissive state, at low temperature, and b) in the transmissive state, at high temperature. Light from a blue LED (peak wavelength at 455 nm), excites the photocured HRI-F27-02 layer that emits light, partly coupled in the planar waveguide and travelling towards the thermoresponsive liquid crystal polymer sensor material. Waveguided light reaching the HRI-RhodB-02 layer is partly absorbed and re-emited as orange-red light. A photodetector measures the red light (632 nm) intensity emitted by this emissive layer as a function of the LCP layer temperature.
